# Supplementary material for: Analysis of the current status and influencing factors of kinesiophobia in tumor patients with peripherally inserted central catheter: A cross-sectional study
Source: PLoS One. 2026 Feb 4;21(2):e0332064. doi: 10.1371/journal.pone.0332064 (PMC12872018; doi:10.1371/journal.pone.0332064)
Supplement: Attachment — Submitted filename: PONE-D-25-33789_reviewer.pdf [file pone.0332064.s003.pdf]

PLOS ONE

Analysis of the current status and influencing factors of kinesiophobia in tumor patients with PICC: A cross-sectional study

--Manuscript Draft--

**Manuscript Number:**	PONE-D-25-33789
**Article Type:**	Research Article
**Full Title:**	Analysis of the current status and influencing factors of kinesiophobia in tumor patients with PICC: A cross-sectional study
**Short Title:**	Kinesiophobia among tumor patients with PICC
**Corresponding Author:**	RONG Li<br>Taizhou Third People's Hoepital<br>Taizhou, Jiangsu CHINA
**Keywords:**	PICC; Tumor; Kinesiophobia; cross-sectional study
**Abstract:**	Background<br>Kinesiophobia exists in tumor patients with PICC, which is ignored by most people. Few studies have explored this phenomenon in depth.Based on the Health Action Process Approach (HAPA) theory, this study aims to explore the current status and influencing factors of kinesiophobia in tumor patients with peripherally inserted central catheter (PICC) after catheterization. The study provides reference for clarifying the mechanism of kinesiophobia and developing nursing intervention plans.<br>Method<br>Through convenience sampling, 162 tumor patients who underwent PICC maintenance in three hospitals in Jiangsu Province from December 4th, 2023, to December 31st, 2024 were selected. The patient general information questionnaire, Tampa scale of kinesiophobia, medical coping modes questionnaire, exercise self-efficacy scale, risk perception questionnaire, outcome expectation scale, exercise intention scale, and social support rating scale were used for evaluation.<br>R esult<br>Tumor patients carrying PICC had a kinesiophobia score of 20.11±6.94 points, and 42.59% of tumor patients with PICC had kinesiophobia. The results of multiple linear regression showed that the duration of catheter placement, pain, exercise self-efficacy, and risk perception are the main influencing factors of kinesiophobia in tumor patients with PICC (P<0.05).<br>Conclusion<br>The incidence of kinesiophobia in tumor patients with PICC is relatively high. Nursing staff should fully evaluate the degree of kinesiophobia and its influencing factors in tumor patients with PICC, and take targeted intervention measures to reduce the incidence and level of kinesiophobia, thereby reducing potential adverse reactions.
**Order of Authors:**	Xiaohua Zhu
	Yan Wu
	RONG Li
	Xiaozhu Qiao
	Ying Yang
	Fang Chen
**Additional Information:**	
**Question**	**Response**
**Financial Disclosure**<br><br>Enter a financial disclosure statement that describes the sources of funding for the work included in this submission. Review	Yes

Powered by Editorial Manager® and ProduXion Manager® from Aries Systems Corporation

the submission guidelines for detailed requirements. View published research articles from *PLOS ONE* for specific examples.<br><br>This statement is required for submission and **will appear in the published article** if the submission is accepted. Please make sure it is accurate.<br><br>**Funded studies**<br>Enter a statement with the following details:<br>• Initials of the authors who received each award<br>• Grant numbers awarded to each author<br>• The full name of each funder<br>• URL of each funder website<br>• Did the sponsors or funders play any role in the study design, data collection and analysis, decision to publish, or preparation of the manuscript?<br><br>Did you receive funding for this work?	
Please add funding details.<br>as follow-up to "**Financial Disclosure**<br><br>Enter a financial disclosure statement that describes the sources of funding for the work included in this submission. Review the submission guidelines for detailed requirements. View published research articles from *PLOS ONE* for specific examples.<br><br>This statement is required for submission and **will appear in the published article** if the submission is accepted. Please make sure it is accurate.	This study was supported by Taizhou "Fengcheng talents" young scientific and technological talents promotion project (2024). Funding bodies did not play any role in this study.

**Funded studies**<br>Enter a statement with the following details:<br>• Initials of the authors who received each award<br>• Grant numbers awarded to each author<br>• The full name of each funder<br>• URL of each funder website<br>• Did the sponsors or funders play any role in the study design, data collection and analysis, decision to publish, or preparation of the manuscript?<br><br>Did you receive funding for this work?"	
Please select the country of your main research funder (please select carefully as in some cases this is used in fee calculation).<br>as follow-up to "**Financial Disclosure**<br><br>Enter a financial disclosure statement that describes the sources of funding for the work included in this submission. Review the submission guidelines for detailed requirements. View published research articles from *PLOS ONE* for specific examples.<br><br>This statement is required for submission and **will appear in the published article** if the submission is accepted. Please make sure it is accurate.<br><br>**Funded studies**<br>Enter a statement with the following details:<br>• Initials of the authors who received each award<br>• Grant numbers awarded to each author<br>• The full name of each funder<br>• URL of each funder website<br>• Did the sponsors or funders play any role in the study design, data collection and analysis, decision to publish, or preparation of the manuscript?<br><br>Did you receive funding for this work?"	CHINA - CN
**Competing Interests**	All authors declare no conflicts of interest.

Use the instructions below to enter a competing interest statement for this submission. On behalf of all authors, disclose any competing interests that could be perceived to bias this work—acknowledging all financial support and any other relevant financial or non-financial competing interests.<br><br>This statement is required for submission and **will appear in the published article** if the submission is accepted. Please make sure it is accurate and that any funding sources listed in your Funding Information later in the submission form are also declared in your Financial Disclosure statement.<br><br>View published research articles from *PLOS ONE* for specific examples.<br><br>**NO authors have competing interests**<br><br>Enter: *The authors have declared that no competing interests exist.*<br><br>**Authors with competing interests**<br><br>Enter competing interest details beginning with this statement:<br><br>*I have read the journal's policy and the authors of this manuscript have the following competing interests: [insert competing interests here]*<br><br>* typeset	
**Ethics Statement**<br><br>Enter an ethics statement for this submission. This statement is required if the study involved:<br><br>• Human participants<br>• Human specimens or tissue<br>• Vertebrate animals or cephalopods<br>• Vertebrate embryos or tissues	This study was approved by the ethics committee of Taizhou Third People's Hospital, approval No.TZSRY-LS-2023YL-026. Before the questionnaire was administered, each participant provided informed consent. This study follows the guidelines set out in the declaration of Helsinki. We also submit them in the attachment.

Powered by Editorial Manager® and ProduXion Manager® from Aries Systems Corporation

• Field research<br><br>Write "N/A" if the submission does not require an ethics statement.<br><br>General guidance is provided below. Consult the submission guidelines for detailed instructions. **Make sure that all information entered here is included in the Methods section of the manuscript.**	

**Format for specific study types**<br><br>**Human Subject Research (involving human participants and/or tissue)**<br>• Give the name of the institutional review board or ethics committee that approved the study<br>• Include the approval number and/or a statement indicating approval of this research<br>• Indicate the form of consent obtained (written/oral) or the reason that consent was not obtained (e.g. the data were analyzed anonymously)<br><br>**Animal Research (involving vertebrate animals, embryos or tissues)**<br>• Provide the name of the Institutional Animal Care and Use Committee (IACUC) or other relevant ethics board that reviewed the study protocol, and indicate whether they approved this research or granted a formal waiver of ethical approval<br>• Include an approval number if one was obtained<br>• If the study involved *non-human primates*, add *additional details* about animal welfare and steps taken to ameliorate suffering<br>• If anesthesia, euthanasia, or any kind of animal sacrifice is part of the study, include briefly which substances and/or methods were applied<br><br>**Field Research**<br><br>Include the following details if this study involves the collection of plant, animal, or other materials from a natural setting:<br>• Field permit number<br>• Name of the institution or relevant body that granted permission	
**Data Availability**<br><br>Authors are required to make all data underlying the findings described fully available, without restriction, and from the time of publication. PLOS allows rare exceptions to address legal and ethical concerns. See the PLOS Data Policy and FAQ for detailed information.	No - some restrictions will apply

Powered by Editorial Manager® and ProduXion Manager® from Aries Systems Corporation

A Data Availability Statement describing where the data can be found is required at submission. Your answers to this question constitute the Data Availability Statement and **will be published in the article**, if accepted.<br><br>**Important:** Stating 'data available on request from the author' is not sufficient. If your data are only available upon request, select 'No' for the first question and explain your exceptional situation in the text box.<br><br>Do the authors confirm that all data underlying the findings described in their manuscript are fully available without restriction?	
**Describe where the data may be found in full sentences. If you are copying our sample text, replace any instances of XXX with the appropriate details.**<br><br>• If the data are **held or will be held in a public repository**, include URLs, accession numbers or DOIs. If this information will only be available after acceptance, indicate this by ticking the box below. For example: *All XXX files are available from the XXX database (accession number(s) XXX, XXX.*).<br>• If the data are all contained **within the manuscript and/or Supporting Information files**, enter the following: *All relevant data are within the manuscript and its Supporting Information files.*<br>• If neither of these applies but you are able to provide **details of access elsewhere**, with or without limitations, please do so. For example:<br><br>*Data cannot be shared publicly because of [XXX]. Data are available from the XXX Institutional Data Access / Ethics Committee (contact via XXX) for researchers who meet the criteria for access to confidential data.*<br><br>*The data underlying the results presented in the study are available from (include the name of the third party*	Data Availability Statement<br>According to reasonable requirements, the corresponding author Li Rong can provide the dataset used and/or analyzed in this study. Email: lirong19900916@163.com

Powered by Editorial Manager® and ProduXion Manager® from Aries Systems Corporation

and contact information or URL).<br>• This text is appropriate if the data are owned by a third party and authors do not have permission to share the data.<br><br>* typeset	
Additional data availability information:	Tick here if your circumstances are not covered by the questions above and you need the journal's help to make your data available.

Powered by Editorial Manager® and ProduXion Manager® from Aries Systems Corporation

Manuscript
Click here to access/download;Manuscript;Title page and manuscript.docx

Analysis of the current status and influencing factors of kinesiophobia in tumor patients with PICC： A cross-sectional study

Xiaohua Zhu[1 ¶], Yan Wu[2¶], Rong Li[2*,#a], Xiaozhu Qiao[3*], Ying Yang[2&], Fang Chen[2&]

[1] Vascular access clinic, Xinghua people's Hospital, No.419, Yingwu South Road, Xinghua City, Taizhou City, Jiangsu Province, China

[2] Vascular access clinic, Taizhou Third People's Hospital, No.98 Chungang East Road, Gaogang District, Taizhou City, Jiangsu Province, China

[3] Nursing Department, Taizhou People's Hospital, No. 366 Taihu Road, pharmaceutical high tech Zone, Taizhou, Jiangsu, China

[#a] Vascular access clinic, Taizhou Third People's Hospital, No.98 Chungang East Road, Gaogang District, Taizhou City, Jiangsu Province, China

Corresponding Author

Email: lirong19900916@163.com(RL)

[¶]These authors contributed equally to this work.

[*]These authors contributed equally to this work.

Abstract

Background

Kinesiophobia exists in tumor patients with PICC, which is ignored by most people. Few studies have explored this phenomenon in depth.Based on the Health Action Process Approach (HAPA) theory, this study aims to explore the current status and influencing factors of kinesiophobia in tumor patients with peripherally inserted central catheter (PICC) after catheterization. The study provides reference for clarifying the mechanism of kinesiophobia and developing nursing intervention plans.

Method

Through convenience sampling, 162 tumor patients who underwent PICC maintenance in three hospitals in Jiangsu Province from December 4th, 2023, to December 31st, 2024 were selected. The patient general information questionnaire, Tampa scale of kinesiophobia, medical coping modes questionnaire, exercise self-efficacy scale, risk perception questionnaire, outcome expectation scale, exercise intention scale, and social support rating scale were used for evaluation.

Result

Tumor patients carrying PICC had a kinesiophobia score of 20.11±6.94 points, and 42.59% of tumor patients with PICC had kinesiophobia. The results of multiple linear regression showed that the duration of catheter placement, pain, exercise self-efficacy, and risk perception are the main

influencing factors of kinesiophobia in tumor patients with PICC ($P<0.05$).

Conclusion

The incidence of kinesiophobia in tumor patients with PICC is relatively high. Nursing staff should fully evaluate the degree of kinesiophobia and its influencing factors in tumor patients with PICC, and take targeted intervention measures to reduce the incidence and level of kinesiophobia, thereby reducing potential adverse reactions.

Introduction

The incidence rate and mortality of malignant tumors in China rank first in the world[1]. Both domestic and international guidelines agree that exercise testing and intervention are generally safe and effective for tumor patients. Proper exercise and physical activity can affect the tumor microenvironment, inhibit cancer cells, enhance immunity, and delay tumor progression of cancer patients[2]. However, recent meta-analyses have shown that 47% of tumor patients experience cancer-related pain[3] [illegible] which directly affects their fear of exercise behavior which [illegible] kinesiophobia[4]. 72.6% of tumor patients require chemotherapy[5], and the peripherally inserted central catheter (PICC), which is inserted through the peripheral vein, has become the most widely used central venous catheter in China due to its minimally invasive nature, avoidance

of repeated punctures, and convenience for safe medication[6,7]. During the period of PICC catheterization, patients should engage in appropriate exercise to reduce the incidence of PICC catheter-associated deep vein thrombosis (CA-DVT)[8]. However, some tumor patients may be concerned about catheter displacement, catheter rupture, and may not be able to engage in physical activity with slightly greater amplitude and frequency during catheterization[9-11]. Compared with other tumor populations, tumor patients carrying PICC have significantly increased fear of exercise, which increases the risk of blood clots and leads to decreased daily living abilities and increased dependence on daily life. Exercise phobia, also known as kinesiophobia, refers to an irrational fear behavior towards exercise and physical activities that manifests in physiological symptoms, psychological discomfort, or fatigue in response to external stimuli[12-13]. At present, research has been conducted on kinesiophobia in populations such as coronary heart disease patients[14], migraine patients[15], and orthopedic postoperative patients[16], exploring the influencing factors. Previous studies on tumor patients with PICC have mostly focused on catheter-related complications, with less attention paid to the side effects caused by reduced exercise. Therefore, this study aims to conduct a cross-sectional observation of tumor patients with PICC to identify the influencing factors of kinesiophobia in tumor patients with PICC, and provide reference for clarifying the mechanism

of kinesiophobia and formulating nursing intervention plans.

Previous theoretical models related to kinesiophobia often used fear avoidance models, which centered around pain. Nowadays, with the expansion of research subjects on kinesiophobia, the existence of fear of exercise suggests not only avoidance of pain, but also other reasons. Considering that kinesiophobia are detrimental to the planning and behavior of tumor patients with PICC, this study intends to adopt the Health Action Process Approach (HAPA) theoretical model to analyze their related factors and underlying influencing mechanisms. Combining stage and continuity characteristics, the model can effectively analyze and predict health behavior and its underlying mechanisms. The main variables include self-efficacy, risk perception, outcome expectations, behavioral intention, planning, and behavior. This theory is suitable for application in this study.

Subject and method

Survey subjects

Convenience sampling was used to select tumor patients who underwent PICC catheter maintenance at three hospitals in Taizhou City, from December 4th, 2023, to December 31st, 2024, as the research subjects. There are a total of 27 variables analyzed in this survey, and the Kendall method is used to take 5 times the number of variables as the required sample size, which is 135 cases. Considering the possibility of missing

sample size, an additional 20% of the sample will be added to ultimately include 162 cases. Inclusion criteria: □Patients diagnosed with malignant tumors by the attending physician; □Age ≥ 18 years old; □First insertion of PICC, with a catheterization time of ≥ 2 weeks; □Can independently complete the questionnaire or complete the questionnaire with the assistance of the researcher. Exclusion criteria: □There are contraindications to exercise; □Merge other serious diseases (such as severe heart, lung, kidney, [illegible] dysfunction, etc.); □Patients with previous mental illnesses or cognitive impairments; □Patients with physical activity disorders.

Investigation tools

General information questionnaire

Based on literature review, the group discussed the design of the questionnaire. The questionnaire includes age, gender, education level, monthly household income, place of residence, time of catheter insertion, PICC placement location (left arm, right arm or other), occurrence of complications (occurrence time), presence of foreign body sensation, pain, and number of intubation attempts.

Tampa scale of kinesiophobia

The Tampa Scale of Kinesiophobia (TSK-11) was used to evaluate tumor patients carrying PICC catheters. This scale was revised and simplified by Woby[17] in 2005 based on the original Tampa Scale of

Kinesiophobia. The revised scale consists of 11 items, divided into three dimensions: activity attitude, activity behavior cognition, and activity behavior. For each item, survey respondents are required to rate their level of agreement, with 1 point for strongly disagree, 2 points for disagree, 3 points for agree, and 4 points for strongly agree. The total score ranges from 11 to 44 points, with higher scores indicating a higher degree of kinesiophobia. The Chinese version of the scale has good internal consistency and test-retest reliability, with a Cronbach'α coefficient of 0.883 and an within-group correlation coefficient (ICC) of 0.798[18]. Jimenez et al.[19] classified fear levels into four levels: no fear (≤ 17 points), mild fear (18-24 points), moderate fear (25-31 points), severe fear (32-38 points), and extreme fear (39-44 points).

Medical coping modes questionnaire

The Medical Coping Modes Questionnaire (MCMQ) revised by Shen Xiaohong[20] was used to evaluate patients' coping modes in various medical situations. The scale consists of 20 items, divided into three dimensions: yielding, avoidance, and facing. The highest score is obtained in one dimension, indicating that patients tend to prefer the coping mode of that dimension.

Exercise self-efficacy scale

The Exercise Self-Efficacy Scale (ESE) designed by Bandura and localized by Tung et al.[21] was used to evaluate tumor patients with

PICC. This scale has been widely used in the assessment of exercise self-efficacy among chronic disease patients abroad. This scale contains a total of 18 items, with each item ranging from 0 to 100 points. A score of 0 indicates complete inability, 50 indicates moderate certainty, and 100 indicates complete certainty. The average score of the 18 items is used as the scale score, and the higher the score, the higher the level of exercise self-efficacy.

Risk perception questionnaire

The survey was conducted using the medical risk perception questionnaire developed by Fang Lei et al[22]. This scale consists of 12 items, divided into three dimensions: economic risk, physical diagnosis and treatment risk, and social psychological risk. It uses the Likert 1-5 scoring method, with 1 point indicating very worried and 5 points indicating not worried at all. The higher the total score of each item, the higher the risk perception.

Exercise outcome expectation questionnaire

The survey was conducted using the exercise outcome expectation scale developed by Renner et al.[23]. This scale includes ten positive outcome expectations and three negative outcome expectations, using the Likert 1-5 scoring method, with 1 point indicating complete impossibility and 5 points indicating complete possibility. Negative outcomes are scored in reverse, and the total score of all items is the outcome

expectation total score. The higher the total score, the higher the positive expectation.

Exercise intention

The survey was conducted using the exercise intention scale developed by Duan[24]. The scale consists of four items, and the survey participants answer the possibilities described in each item using the Likert 1-5 scoring method. One point is complete impossibility, and five points are complete possibility. The exercise intention is determined by the total score of each item, and the higher the score, the higher the exercise intention.

Social support rating scale

The Social Support Rating Scale (SSRS) developed by Xiao Shuiyuan et al.[25] was used for evaluation. This scale consists of 10 items, divided into three dimensions: objective support, subjective support, and support utilization. It uses a Likert 1-4 point scoring method, with 1 point indicating strongly disagree and 4 points indicating strongly agree. The higher the total score, the higher the level of social support.

Data collection and quality control

Two nurses who regularly work in the vascular access clinic were trained before the investigation. After passing the training assessment, the two nurses took turns collecting data. During the survey, a unified guiding language was used to inform patients of the research purpose and

questionnaire filling method, and the questionnaire was distributed after obtaining informed consent. During the filling process, no hints was given. If patients need assistance with questionnaire filling, the investigator explained it through unified explanatory words. After completing the questionnaire, it was collected immediately. One researcher transcribed it into electronic data within 48 hours, and the other verified it.

Statistical methods

SPSS 26.0 was used for data processing. Pearson correlation analysis was used between kinesiophobia and various variables, independent sample t-test or chi-square test was used for univariate analysis, binary logistic regression analysis was used for multivariate analysis.

Ethical statement

This study was approved by the ethics committee of Taizhou Third People's Hospital, approval No.TZSRY-LS-2023YL-026. Before the questionnaire was administered, each participant provided written informed consent. This study follows the guidelines set out in the declaration of Helsinki.

Results

Questionnaire results on kinesiophobia, medical coping

modes, and exercise self-efficacy from tumor patients with PICC catheterization

The patient's kinesiophobia score was 20.11±6.94 points, and the incidence of kinesiophobia was 42.59% (69 cases). Among them, activity attitude was 4.82±2.56 points, activity cognition was 10.67±2.94 points, and activity behavior wad 5.20±1.69) points. 54 cases (33.33%) tended to "facing", 46 cases (28.40%) tended to "avoidance", and 62 cases (38.27%) tended to "yielding". The exercise self-efficacy score was 58.04±18.79 points. The risk perception score was 25.57±8.37 points. The exercise outcome expectation score was 29.31±8.67 points. The exercise intention score was 12.80±4.03 points. The social support score is 32.02±13.16 points.

Comparison of kinesiophobia scores in tumor patients with PICC catheterization with different characteristics is shown in Table 1.

Table 1. Comparison of Kinesiophobia Scores in Tumor Patients With PICC Catheterization With Different Characteristics (x±s)

Item	Number of cases	TSK score	*t/F*	*P*
Age (years old)			0.388	0.699
18~60	53	20.12±6.12		
＞60	109	19.96±7.33		
Gender			-1.515	0.132

Male	77	19.25±7.14		
Female	85	20.89±6.70		
Degree of education			7.606	0.001
Elementary school and below	68	22.51±7.31		
Junior and senior high school	59	18.34±6.34		
University and above	35	18.43±5.87		
Monthly household income			0.125	0.882
≤5000	40	20.58±7.87		
5000-10000	54	19.87±6.46		
＞10000	68	20.03±6.82		
Place of residence			-2.300	0.023
Town	83	18.90±7.33		
Rural area	79	21.38±6.31		
Placement duration			9.260	＜0.001
≤1 month	37	25.03±8.86		
1-3 months	41	18.73±6.43		
3-6 months	41	18.71±5.26		
＞6 months	43	18.53±4.92		

Catheter location			3.024	0.051
Left upper arm	64	21.75±7.83		
Right upper arm	94	19.04±6.03		
Other parts	4	19.00±8.72		
Complications occurrence			7.001	＜0.001
Yes	23	28.35±5.18		
No	139	18.75±6.23		
Foreign body sensation			5.319	＜0.001
Yes	87	22.57±6.91		
No	75	17.25±5.83		
Pain			6.679	＜0.001
Yes	26	27.50±8.08		
No	136	18.70±5.73		
Number of intubation attempts			-1.053	0.304
≤1 time	143	19.86±6.70		
＞1 time	19	22.00±8.52		
Medical coping modes			5.816	0.004
Yielding	62	19.56±7.45		

Avoidance	46	22.87±6.28
Facing	54	18.39±6.24

Correlation analysis between kinesiophobia and pain, exercise self-efficacy, risk perception, outcome expectation, exercise intention, social support in tumor patients with PICC

The Pearson correlation analysis results showed that kinesiophobia was positively correlated with pain (r=0426, P<0.01), positively correlated with risk perception (r=0.684, P<0.01), negatively correlated with exercise self-efficacy (r=-0.677, P<0.01), negatively correlated with outcome expectation (r=-0.550, P<0.01), negatively correlated with exercise intention (r=-0.524, P<0.01), and negatively correlated with social support (r=-0.511, P<0.01). See Table 2 for details.

Table 2 Correlation Between TSK-11 Scores of Patients With PICC Catheterization and Various Variables (*N*=162)

Items	TSK-11	ESE	Risk perception	Outcome expectation	SSRS	Pain score	Exercise intention
TSK-11	1						
ESE	-.677**	1					
Risk perception	.684**	-.662**	1				

Outcome expectation	-.550**	.525**	-.767**	1			
SSRS	-.511**	.470**	-.746**	.655**	1		
Pain score	.426**	-.294**	.244**	-.187*	-0.15	1	
Exercise intention	-.524**	.485**	-.688**	.606**	.593**	-.213**	1

**When *P*<0.01, the correlation is significant;* when *P*<0.05, the correlation is significant.

Multiple linear regression analysis of kinesiophobia in tumor patients with PICC

Multiple linear regression was performed with the TSK-11 score of tumor patients with PICC as the dependent variable and the variables with statistically significant differences in univariate analysis as the independent variables (αin=0.05, αout=0.10). The assignment method for categorical variables is shown in Table 3, while the remaining variables are inputted with their original values. The results of multiple linear regression analysis showed that pain, exercise self-efficacy, risk perception level, and catheter insertion duration were factors affecting kinesiophobia in patients with PICC, as shown in Table 4.

Table 3 Categorical Variable Assignment Method

**Independent**	**Assignment**

**variables**	
Degree of education	Elementary school and below=1; Junior and senior high school=2; University and above=3
Place of residence	Town=1; Rural area=2
Placement duration	≤1 month=1; 1-3 months=2; 3-6 months=3; ＞6 months=4
Complication	No=0, Yes=1
Foreign body sensation	No=0, Yes=1
Coping modes	Yielding=1; Avoidance=2; Facing=3
Pain	No=0, Yes=1

Table 4 Results of Multiple Linear Regression Analysis on Factors Influencing Kinesiophobia in Tumor Patients with PICC (*N*=162)

**Independent variable**	**Regression coefficient**	**Standard error**	**Standardized regression coefficient β**	***t*-value**	***P*-value**
Constant	20.122	5.388	-	3.735	＜0.001
Pain	3.065	1.156	0.163	2.652	0.009
PICC carrying duration	-1.248	0.356	-0.200	-3.506	0.001
Risk perception level	0.279	0.088	0.336	3.157	0.002
ESE	-0.096	0.025	-0.261	-3.891	＜0.001

Note: R^2=0.674; R^2=0.645; F=23.504; P<0.001

Discussion

Patients with PICC have a higher incidence of kinesiophobia, but the severity is relatively mild

This study found that 42.59% of tumor patients with PICC had kinesiophobia, but the overall score (20.11±6.94 points) was mainly mild fear. This result is lower than previous studies on kinesiophobia in patients with chronic diseases, such as the 53% (95% CI: 37-68%) incidence of kinesiophobia in patients with coronary heart disease[26], but higher than some orthopedic disease patients[27, 28]. The higher incidence of kinesiophobia in patients with PICC may be related to their special medical situations. On the one hand, the risk of catheter-related complications (such as displacement, rupture, thrombosis) increases patients' concerns about exercise. On the other hand, the physical weakness, pain, and psychological pressure caused by the disease and treatment (such as chemotherapy and radiotherapy) in tumor patients further exacerbate their tendency to avoid exercise[29]. In the daily management of PICC, medical staff should conduct a comprehensive evaluation of patients and communicate fully. Once the patient's mistaken perception of movement or activity causing catheter dysfunction leading to signs of kinesiophobia is identified, it should be corrected immediately.

Nursing staff need to pay attention to health education and the dissemination of knowledge related to kinesiophobia during PICC catheterization, and provide education or demonstration through multiple channels and forms to improve follow-up management. Comprehensive management should be carried out based on whether the patient has kinesiophobia and their grading level, such as sharing exercise experience, improving self-efficacy, and forming positive interactions.

Analysis of factors affecting kinesiophobia in tumor patients with PICC

Pain

Pain is a significant predictor of kinesiophobia (β=0.163, P=0.009), which is consistent with the core viewpoint of the fear avoidance model[30, 31] and the findings of Wang[32]. In this study, patients with pain had significantly higher TSK-11 scores than those without pain (27.50±8.08 vs. 18.70±5.73). This result can be explained by a dual mechanism: firstly, pain directly triggers patients' expectations of increased pain induced by exercise, forming a negative cycle of "exercise-pain"; secondly, pain activates psychological avoidance mechanism and reduces patients' motivation to participate in exercise. It is worth noting that about 32% of tumor patients face the dilemma of inadequate treatment for cancer-related pain[33]. Especially for tumor patients, the sources of pain are complex, such as catheter stimulation,

tumor infiltration, or treatment side effects. Therefore, in clinical practice, priority should be given to distinguishing pain types and adopting multimodal analgesia strategies. For example, combining pharmacological analgesia (e.g. opioid drugs) with non-pharmacological interventions (e.g. physical therapy or cognitive-behavioral therapy) can effectively alleviate the inhibitory effect of pain on exercise behavior.

Catheterization duration

The duration of catheter placement is negatively correlated with kinesiophobia ($\beta=-0.200$, $P=0.001$), and patients with catheter placement ≤ 1 month have significantly higher TSK-11 scores than those with long-term catheterization (25.03±8.86 vs. 18.53±4.92). This result supports the viewpoint of “behavioral stage change” in the Health Action Process Orientation (HAPA) theory: as the duration of catheterization increases, patients gradually transition from the “intention stage” to the “action and maintenance stage”, accumulating successful experience through repeated practice (e.g. daily activities, catheterization care), thereby enhancing confidence in exercise safety. In addition, patients with long-term catheterization may have received more systematic education on catheter maintenance. For example, Anna et al.’s research[34, 35] suggests that regular follow-up and health education can help patients establish an objective understanding of catheter stability and reduce fear caused by “unknown risks”. However, the high level of kinesiophobia in

the early stages of catheterization suggests the need for strengthened early intervention, such as using a progressive exercise plan (e.g. transitioning from low-intensity walking to upper limb activities) to help patients adapt to the presence of the catheter and establish adaptive behavior patterns.

Risk perception level

The positive predictive effect of risk perception on kinesiophobia is strong (β=0.336, P=0.002), which is consistent with the hypothesis of "risk perception driving health behavior decision-making" in the HAPA theory. In this study, patients' concerns about catheter-related complications (e.g. thrombosis, infection) and economic burden significantly increased their tendency towards kinesiophobia. Paras-Bravo et al.'s qualitative research further revealed[9, 36] that patients often restrict their activities and even avoid necessary household chores due to the imagination of "catheter rupture and displacement". This phenomenon suggests that patients may have cognitive biases in their risk perception, overestimating the risk of low probability events. Clinical interventions require targeted correction of such biases, for example, helping patients establish a rational understanding of catheter safety through visualization tools (e.g. catheter structure model demonstrations) or successful case sharing. In addition, Fang Lei's research emphasizes that risk communication strategies need to be combined with patients' cultural backgrounds, such as using more

intuitive educational methods (e.g. graphic manuals or videos) for rural patients to reduce their excessive concerns about economic risks.

Exercise self-efficacy

Exercise self-efficacy (ESE) is a key protective factor for kinesiophobia in tumor patients with PICC ($\beta=-0.261$, $P<0.001$). In this study, individuals with lower ESE scores had higher levels of kinesiophobia, suggesting that self-efficacy may indirectly affect exercise behavior intention by regulating individual confidence in exercise ability. Li et al.[37] (small group scenario simulation training) also found in their study of tumor patients with PICC that high self-efficacy can significantly alleviate the limitations of daily activities caused by kinesiophobia. Therefore, nursing interventions should focus on enhancing patients' self-efficacy. Specific strategies for healthcare professionals may include but are not limited to: □goal setting and decomposition, such as breaking down "walking for 10 minutes a day" into "initial 5 minutes and gradually increasing"; □peer support program: encouraging patients to exchange experiences with patients who have successfully adapted to exercise; □ enhancing the successful experience by providing immediate feedback (such as exercise records) to strengthen the patient's sense of achievement. Research shows that the above nursing complementary therapy can help patients improve their self-efficacy and management ability[38]. In addition, there is a synergistic effect between social

support and self-efficacy. Ye et al.[39] pointed out that emotional encouragement and practical assistance from family members (such as accompanying exercise) can further enhance patients' exercise confidence and tolerance.

Research limitations and prospects

This study has the following limitations. Firstly, the cross-sectional design cannot infer causal relationships between variables, for example, risk perception and kinesiophobia may be mutually causal. Secondly, the samples all come from three hospitals in the same region, which may lead to selection bias. Future research needs to expand to tumor patients in multiple regions and disease types. Finally, the lack of inclusion of biomarkers (such as inflammatory factors) in analyzing the physiological mechanisms of kinesiophobia limits the comprehensiveness of the results. Future research directions include: □using longitudinal design to track the dynamic changes of kinesiophobia, especially the intervention effects at different time points after catheterization; □combining mixed methods research (quantitative+qualitative) to deeply explore patients' subjective experiences and cultural influencing factors; □developing digital intervention tools (such as mobile applications) based on HAPA theory, and achieving personalized management through phased goal setting, risk perception correction, and effectiveness improvement modules. At the practical level, it is recommended to develop a phased nursing plan: focus

on managing pain and risk perception in the early stages of catheterization (such as multidisciplinary pain group intervention), and strengthen exercise self-efficacy and social support in the later stages (such as family-medical team collaboration plans) to systematically reduce the incidence of kinesiophobia and improve patients' quality of life.

Conclusion

This study shows that the incidence of kinesiophobia in tumor patients with PICC is relatively high, but the degree is mild. Kinesiophobia in tumor patients with PICC is influenced by multiple factors such as pain, duration of catheter placement, risk perception, and exercise self-efficacy. Clinical nursing needs to combine individualized assessment and theory-driven intervention to promote safe patient participation in exercise through pain management, cognitive remodeling, and efficacy enhancement. Further exploration of multidimensional intervention strategies is needed in the future to achieve a behavioral shift from "fear avoidance" to "active adaptation", ultimately improving the long-term prognosis and quality of life of tumor patients.

Acknowledgments

This study was supported by Taizhou "Fengcheng talents" young

scientific and technological talents promotion project (2024). Funding bodies did not play any role in this study.

Data Availability Statement

According to reasonable requirements, the corresponding author Li Rong can provide the dataset used and/or analyzed in this study.

Statement

All authors declare no conflicts of interest.

Author Contributions

Project preparation, conceptualization, methodology, software,Writing - Initial Draft: Rong Li

Data Collection and Organization: Xiaohua Zhu, Yan Wu, Xiaozhu Qiao

Supervision: Ying Yang and Fang Chen

Software, verification: Xiaohua Zhu

Writing - Commentary and Editing: Yan Wu and Xiaozhu Qiao

All authors have full access to all data in the study, and the corresponding author bears ultimate responsibility for the submission decision. The corresponding author confirms that all listed authors meet the attribution criteria and no other eligible authors have been omitted.

References

[1] Sung H, Ferlay J, Siegel R L, et al. Global Cancer Statistics 2020: GLOBOCAN Estimates of Incidence and Mortality Worldwide for 36 Cancers in 185 Countries[J]. CA Cancer J Clin, 2021,71(3):209-249.

[2] Cong Minghua, Shi Hanping. Consensus of Chinese Experts on Exercise Therapy for Cancer Patients[J]. Scientia Sinica(Vitae), 2022, 52 (04): 587-602.

[3] Haenen V, Evenepoel M, De Baerdemaecker T, et al. Pain prevalence and characteristics in survivors of solid cancers: a systematic review and meta-analysis[J]. Support Care Cancer, 2022,31(1):85.

[4] Rogers A H, Farris S G. A meta-analysis of the associations of elements of the fear-avoidance model of chronic pain with negative affect, depression, anxiety, pain-related disability and pain intensity[J]. Eur J Pain, 2022,26(8):1611-1635.

[5] Prospective Industry Research Institute. 2020 Analysis of the Development Status of the Global and Chinese Oncology Drug Industry: Huge Growth Potential in Medical Demand for Domestic Cancer Patients [EB/OL]. (2020-11-05)[0011-05-01]. https://bg.qianzhan.com/report/detail/300/201105-d74e0f94.html.

[6] Wang Lei, Nie Shengxiao, Sun Hong. A Study on the Current Status of Intravenous Treatment in 743 Secondary and Tertiary Hospitals[J]. Chinese Journal of Modern Nursing, 2020, 26 (32): 4494-4500.

[7] Mielke D, Wittig A, Teichgraber U. Peripherally inserted central venous catheter (PICC) in outpatient and inpatient oncological treatment[J]. Support Care Cancer, 2020,28(10):4753-4760.

[8] Gorski L A, Hadaway L, Hagle M E, et al. Infusion Therapy Standards of Practice, 8th Edition[J]. J Infus Nurs, 2021,44(1S Suppl 1):S1-S224.

[9] Paras-Bravo P, Paz-Zulueta M, Santibanez M, et al. Living with a peripherally inserted central catheter: the perspective of cancer outpatients-a qualitative study[J]. Support Care Cancer, 2018,26(2):441-449.

[10] Shao Jie, Xiong Yu, Ma Dan, et al. Meta-synthesis of Qualitative Research on Fear of Patients Related to PICC Catheterization[J]. Journal of Nursing Science, 2022,37(17):80-82.

[11] Cui Bo, Fu Rong, Zhang Ziyan. System Evaluation and Meta Integration on Real Experience of Patients Using PICC Catheter[J]. Chinese Nursing Research, 2018, 32 (11): 1773-1778.

[12] Kori S. Kinesphobia : a new view of chronic pain behaviour[J]. Pain Management, 1990.

[13] Knapik A, Saulicz E, Gnat R. Kinesiophobia - introducing a new diagnostic tool[J]. J Hum Kinet, 2011,28:25-31.

[14] Keessen P, Latour C, van Duijvenbode I, et al. Factors related to fear of movement after acute cardiac hospitalization[J]. BMC Cardiovasc Disord, 2020,20(1):495.

[15] Pinheiro C F, Bevilaqua-Grossi D, Florencio L L, et al. Is kinesiophobia related to fear of falling, dizziness disability, and migraine disability in patients with migraine?[J]. Physiother Theory Pract, 2022,38(13):2727-2735.

[16] Aydemir B, Huang C H, Foucher K C. Strength and physical activity in osteoarthritis: The mediating role of kinesiophobia[J]. J Orthop Res, 2022,40(5):1135-1142.

[17] Woby S R, Roach N K, Urmston M, et al. Psychometric properties of the TSK-11: a shortened version of the Tampa Scale for Kinesiophobia[J]. Pain, 2005,117(1-2):137-144.

[18] Cai L, Liu Y, Woby S R, et al. Cross-Cultural Adaptation, Reliability, and Validity of the Chinese Version of the Tampa Scale for Kinesiophobia-11 Among Patients Who Have Undergone Total Knee Arthroplasty[J]. J Arthroplasty, 2019,34(6):1116-1121.

[19] Jimenez-Cebrian A M, Becerro-de-Bengoa-Vallejo R, Losa-Iglesias M E, et al. Kinesiophobia Levels in Patients with Parkinson's Disease: A Case-Control Investigation[J]. Int J Environ Res Public Health, 2021,18(9).

[20] Shen Xiaohong, Jiang Qianjin. Report on Application of Chinese Version of MCMQ in 701 Patients[J]. Chinese Journal of Behavioral Medical and Brain Science, 2000 (01): 22-24.

[21] Tung W, Gillett P A, Pattillo R E. Applying the Transtheoretical Model to physical activity in family caregivers in Taiwan[J]. Public Health Nurs, 2005,22(4):299-310.

[22] Fang Lei. The Study of Questionnaire Construction of Risk Perception and It's Influence Factors for Common Chronic Inpatients[D]. College of Nursing, Fourth Military Medical University, 2015.

[23] Renner B, Schwarzer R. Risk and health behaviors: documentation of the Scales of the Research Project "Risk Appraisal Consequences in Korea"(RACK)[J]. Risk and Health Behaviors: Documentation of the Scales of the Research Project "Risk Appraisal Consequences in Korea"(RACK), 2005:1-55.

[24] Duan Y. Stage models of physical activity behavior and their application to Chinese adults[D]. University of Bayreuth Press, 2006.

[25] Xiao Shuiyuan. Theoretical Basis and Research Application of the Social Support Rating Scale[J]. Journal of Clinical Psychiatry, 1994 (2): 98-100.

[26] Rong L, Zhang G, Ren Z, et al. Prevalence and the Associated Factors of Kinesiophobia among Patients with Coronary Artery Disease: A Systematic Review and Meta-Analysis[J]. Int J Gerontol, 2024,18(1):2-8.

[27] Wu Hongya, Feng Shuo, Liu Huizi, et al. Analysis of Influencing Factors of Postoperative Kinesiophobia in Patients with Osteoporotic Vertebral Compression Fractures[J]. Chinese Medical

Innovations, 2023,20(09):147-151.

[28] Xu Huiping, Zhang Yangai, Liu Yanjin, et al. A Multifactorial Model of Kinessiophobia in Patients with Knee Arthroplasty[J]. Chinese Journal of Nursing, 2021, 56 (10): 1460-1465.

[29] Wang Y, Qiu X. Kinesiophobia and related factors in cancer patients with TIAPs during the long term: a cross-sectional survey[J]. Support Care Cancer, 2022,30(6):4927-4934.

[30] Bullock G S, Sell T C, Zarega R, et al. Kinesiophobia, Knee Self-Efficacy, and Fear Avoidance Beliefs in People with ACL Injury: A Systematic Review and Meta-Analysis[J]. Sports Med, 2022,52(12):3001-3019.

[31] Alpalhao V, Cordeiro N, Pezarat-Correia P. Kinesiophobia and Fear Avoidance in Older Adults: A Systematic Review on Constructs and Related Measures[J]. J Geriatr Phys Ther, 2022,45(4):207-214.

[32] Liuyue W, Juxin G, Chunlan H, et al. Status and influencing factors of patients with kinesiophobia after insertion of peripherally inserted central catheter: A cross-sectional study[J]. Medicine (Baltimore), 2022,101(30):e29529.

[33] Zhang H. Cancer Pain Management-New Therapies[J]. Curr Oncol Rep, 2022,24(2):223-226.

[34] Henriksson A, Johansson B, Radu C, et al. Is it safe to exercise during oncological treatment? A study of adverse events during endurance and resistance training - data from the Phys-Can study[J]. Acta Oncol, 2021,60(1):96-105.

[35] Liu A, Liu X, Li Z, et al. Effect of exercise therapy on PICC-associated venous thromboembolism: A meta-analysis[J]. Phlebology, 2023,38(2):103-114.

[36] Ryan C, Hesselgreaves H, Wu O, et al. Protocol for a systematic review and thematic synthesis of patient experiences of central venous access devices in anti-cancer treatment[J]. Syst Rev,

2018,7(1):61.

[37] Li Bingshi, Hou Qingmei. Effect of Small Group Scenario Simulation Training on Self-care Ability and Self-efficacy among Tumor Patients with Peripherally Inserted Central Catheter[J]. Nursing Journal of Chinese People's Liberation Army, 2016, 33 (13): 28-31.

[38] Onishi K. Exploring the role of complementary therapy in enhancing quality of life for cancer survivors: A nursing perspective. *Asia Pac J Oncol Nurs*. 2024;11(8):100541.

[39] Ye F, Wu Y. Impacts of self-confidence cultivation combined with family collaborative nursing on the hope level, stigma and exercise tolerance in patients undergoing radical resection of pulmonary carcinoma[J]. Front Surg, 2023,10:1095647.

Click here to access/download
Supporting Information - Compressed/ZIP File Archive
Basic data and analysis.zip
