# Supplementary material for: Analysis of the current status and influencing factors of kinesiophobia in tumor patients with peripherally inserted central catheter: A cross-sectional study
Source: PLoS One. 2026 Feb 4;21(2):e0332064. doi: 10.1371/journal.pone.0332064 (PMC12872018; doi:10.1371/journal.pone.0332064)
Supplement: Attachment — Submitted filename: PLOS.docx [file pone.0332064.s001.docx]

A literature review is present, but the gap is not clearly specified.

How HAPA theory was integrated into the study (e.g., variable selection) is not explained.

A clear research hypothesis should be added.

Missing data management is not explained.

Both ‘binary logistic regression’ and ‘multiple linear regression’ are mentioned – this is inconsistent.

It should be stated whether regression assumptions were tested.

The inclusion of variables in the model should be justified.

The discussion section is quite long and repetitive.

It contains a summary rather than an interpretation of the findings.

Concrete recommendations based on the findings: pain and risk perception management in the early stages, self-efficacy-enhancing education programmes in the later stages.

A stronger nursing-focused discussion should be provided.

Terminology should be standardised.

Sentence structure should be simplified.

Patients' cancer types may also contribute to the development of kinesiophobia. It would be appropriate to add information about patients' disease duration and knowledge about cancer.

References to studies on kinesiophobia related to different cancers could be added. E.g.:

Malchrowicz-Mośko, E., Nowaczyk, P., Wasiewicz, J., Urbaniak, T., Siejak, W., Rozmiarek, M., ... & Castañeda-Babarro, A. (2023). The level of kinesiophobia in breast cancer women undergoing surgical treatment. *Frontiers in Oncology*, *13*, 1010315.

Cihan, E., Karalezli, I., Pirincci, C. S., Cavdar, O. F., Goger, Y. E., Aytar, A., & Karalezli, G. (2025). Physical activity, fatigue, kinesiophobia and quality of life: comparative study of prostate cancer survivors with healthy controls. *BMJ Supportive & Palliative Care*, *15*(2), 213-220.

Malchrowicz-Mośko, E. (2022). Kinesiophobia among breast cancer survivors one year after hospital treatment. *International Journal of Environmental Research and Public Health*, *19*(21), 14565.
