# Supplementary material for: Analysis of the current status and influencing factors of kinesiophobia in tumor patients with peripherally inserted central catheter: A cross-sectional study
Source: PLoS One. 2026 Feb 4;21(2):e0332064. doi: 10.1371/journal.pone.0332064 (PMC12872018; doi:10.1371/journal.pone.0332064)
Supplement: Attachment — Submitted filename: Response to Reviewers.doc [file pone.0332064.s002.doc]

**Dear Editor and Reviewers,**

Thank you very much for giving us opportunities to revise our manuscript, and we appreciate the reviewers and editor a lot for your positive and constructive comments and suggestions. We have studied your comments carefully and have made revisions which are marked in red in the “Revised Manuscript With Track Changes”. We hope the corrections will meet with your approval.

**Editor**

1 Thank you for submitting your manuscript to PLOS ONE. After careful consideration, we feel that it has merit but does not fully meet PLOS ONE’s publication criteria as it currently stands. Therefore, we invite you to submit a revised version of the manuscript that addresses the points raised during the review process.

Response: We sincerely appreciate the opportunity to revise our manuscript. We have carefully incorporated into the manuscript. We have also reformatted the manuscript to comply with the journal's guidelines.

2 The manuscript has been evaluated by two reviewers, and their comments are available below.The reviewers have raised a number of concerns that need attention. In particular, they request additional information on the methodological and statistical aspects of the study.Could you please revise the manuscript to carefully address the concerns raised?

Response: We are also truly grateful for the constructive comments from the reviewers. We have thoroughly revised our manuscript based on these insights and hope that it now meets the required standards.

3 If applicable, we recommend that you deposit your laboratory protocols in protocols.io to enhance the reproducibility of your results.

Response: Thank you for this suggestion. Our cross-sectional survey design utilized validated questionnaires rather than wet-lab experiments, so a [protocols.io](https://protocols.io/) deposit is not typical for this methodology. The full details are provided in the Methods section. We are, however, happy to provide the complete survey package (all scales and instruments) to interested researchers upon request to ensure full transparency.

4 We note that you have indicated that there are restrictions to data sharing for this study. For studies involving human research participant data or other sensitive data, we encourage authors to share de-identified or anonymized data. However, when data cannot be publicly shared for ethical reasons, we allow authors to make their data sets available upon request.

Response: We thank the editor for this important reminder. In accordance with the journal's policy and to uphold our ethical commitment to participants, we have obtained approval from our Institutional Ethics Committee to share a de-identified version of the dataset. Subsequently, we have deposited the de-identified data in the Science Data Bank (Science DB) repository, and the dataset will be made accessible upon reasonable request. The data files are available in the Science DB: <https://www.scidb.cn/anonymous/YXllSVpu.> (The corresponding accession number/DOI will be provided during the production stage if the manuscript is accepted for publication.)

5 We note that the grant information you provided in the ‘Funding Information’ and ‘Financial Disclosure’ sections do not match.

Response: Our project was supported by two distinct grants: the 2024 Taizhou "Fengcheng Talents Program" Young Science and Technology Talent Support Project (which does not carry a specific grant number and is designated on an annual basis) and the Jiangsu Provincial Young Science and Technology Talent Support Project (Grant No. JSTJ-2024-624).

The initial inconsistency in the grant information submission arose because the "Fengcheng Yingcai Program" lacks a formal project number and is identified by its annual title, which posed challenges during the initial online submission process. Both funding sources are talent cultivation programs and do not involve any conflicts of interest.

6 We note that there is identifying data in the Supporting Information file <Basic data and analysis.zip>.

Response: We thanks for the valuable reminder. As recommended, we have revised the manuscript to remove all potentially identifiable patient characteristics from the baseline data table, such as gender, education level, income, residence, and PICC insertion site, to uphold strict ethical standards for anonymity. We are grateful for your expert oversight.

7 Please include captions for your Supporting Information files at the end of your manuscript, and update any in-text citations to match accordingly.

Response: We thank the editor for this guidance. After careful review, we believe that all essential data and materials necessary to support the findings of our study are already presented within the main manuscript. We are not currently submitting any separate supporting information files.

However, should the editorial team feel that specific additional data or materials would strengthen the publication, we would be grateful for further clarification on what type of supporting information is recommended. We are fully prepared to comply with any specific requests.

**Reviewer 1**

1 Abstract:
1.1 Introduction: Remove the first and second sentences as it distracts from the main focus of the study. Instead, you may briefly define the catheter or explain the reason for its use.
Response: Thank you for your pertinent suggestions. We have deleted the first sentence and added the background of PICC catheter usage as suggested. We added: *“Peripherally Inserted Central Catheters (PICCs) are widely utilized in tumor patients due to their lower risk of complications, extended indwelling duration, reduced local tissue trauma, and overall cost-effectiveness.”*

1.2 Results: Report the significance levels (e.g., p, r) appropriately and clearly indicate which findings are statistically significant.
Response: Thank you for your reminder. We have added the values of r and P in the results section. Should our interpretation be inaccurate, we would be grateful for further clarification.

1.3 Conclusion: Do not repeat statements that belong in the results section. The conclusions should be clear, concise, and supported by clinical implications.
Response: We are grateful for the reviewer's valuable input. The manuscript has been modified based on this feedback, and we would be grateful for any further advice should further refinement be needed. The result of our modification was as follows: *“The findings underscore a significant clinical concern regarding kinesiophobia among tumor patients with PICC. It is essential for nursing staff to implement systematic assessments and tailored interventions aimed at mitigating kinesiophobia. Addressing this issue can contribute to reducing associated adverse reactions and improving patient mobility and overall quality of life.”*

1.4 Keywords: Where are your keywords?
Response: Very sorry, we forgot the keywords due to negligence. Thank you very much for your reminder. The keywords are: Peripherally Inserted Central Catheter; Tumor; Kinesiophobia; Cross-Sectional Study; Exercise Self-efficacy; Health Action Process Approach theoretical.

2 Introduction:
2.1 Review and revise according to proper academic writing conventions. The use of headings is currently inappropriate.
Response: Thanks a lot. We have also made modifications to the beginning and made detailed changes to the "Introduction" section again. We have reorganized the structure and refined the sentence structure, hoping to fully comply with academic writing norms and requirements.

2.2 Add a reference for the sentence beginning with: “Previous studies on tumor patients with PICC have mostly focused on catheter-related complications...”
Response: Thanks a lot. It is indeed necessary to add references here, and we have done so.

2.3 In the final paragraph, for the sentence:
“Previous theoretical models related to kinesiophobia often used fear avoidance models, which centered around pain. Nowadays, with the expansion of research subjects on kinesiophobia...”,
include the following reference:

Physical activity, fatigue, kinesiophobia and quality of life: comparative study of prostate cancer survivors with healthy controls. 2025;15:213-220. https://doi.org/10.1136/spcare-2024-005239
Response: We appreciate the reviewer's valuable suggestion. Accordingly, we have incorporated the recommended reference into the relevant section.

3 Methods:

3.1 Please provide a proper reference for the Kendall method and explain the rationale for its use in your study.

Response: We sincerely appreciate the reviewer's valuable feedback regarding our sample size estimation. We have now included references for the use of the Kendall method. While the rule of using five times the number of variables is an empirical approach, it is generally considered acceptable in cases where well-established scales with clear factor structures are used and when high data quality with good participant compliance is expected. We fully acknowledge the reviewer's valid concern that this method lacks statistical rigor compared to more formal power-based calculations. We will be sure to adopt a more systematic and rigorous sample size calculation in our future studies. Thank you again for this constructive suggestion.

3.2 Is the Kendall method a statistical analysis method or a method specific to osteoarthritis? Based on this, how were you able to determine your sample?
Response: Thank you for your guidance. Kendall is a widely used sample size estimation method at home and abroad. The Kendall method we use is a statistical approach. We calculated based on 5 times the independent variable. There are 11 independent variables in the general data, TSK-11 has 3 dimensions, MCMQ has 3 dimensions, ESE has 1 dimension, Risk perception questionnaire has 3 dimensions, Exercise Results Questionnaire has 1 dimension, Exercise Intention has 2 dimensions, SSRS has 3 dimensions, a total of 27 analytical variables. Five times that is 135 cases.
3.3 Why was no power analysis conducted? Is the sample size sufficient? Consider including a post-hoc power analysis.

Response: We sincerely thank the reviewer for raising this important methodological point. A post-hoc power analysis based on the observed effect sizes indicated that a total sample of 323 would be ideal. Our final sample of 162 participants was initially determined by a widely cited empirical rule of thumb in similar clinical survey studies, which suggests a target of 5-10 participants per predictor variable. While we fully acknowledge that the achieved sample size is lower than the post-hoc calculation suggests, we would be grateful if the reviewer could consider that this empirical approach, while less precise than an a priori power analysis, is a recognized and pragmatic method in exploratory clinical research, particularly under constraints of time and patient accessibility. Importantly, despite the smaller sample, the key predictors in our regression model demonstrated substantial and statistically significant effects. We have transparently acknowledged the sample size limitation in the revised manuscript and recommend future validation with larger, prospectively powered studies.

Z^2^=1.96*1.96=3.8416; p=0.30; δ=0.05

N=323

[1] Gencay Can A, Can SS, Ekşioğlu E, Çakcı FA. Is kinesiophobia associated with lymphedema, upper extremity function, and psychological morbidity in breast cancer survivors?. *Turk J Phys Med Rehabil*. 2018;65(2):139-146.

3.4 Justify your use of logistic regression in the statistical analysis section.

Response: We appreciate the reviewer for pointing out this discrepancy. The analysis has been correctly conducted using multiple linear regression, not binary logistic regression. We have revised the manuscript accordingly to ensure the statistical methods are accurately described throughout.
3.5 Please place the ethics section after the Acknowledgments section, rather than within the main text of the manuscript.

Response: Thank you for the opportunity to clarify. We note that the ethical statement is presented within the main text of many recent publications in your journal. Following this precedent, we included it in the main body of our manuscript. We sincerely apologize if this does not align with the specific requirements for our submission and humbly submit that we can move it to another section immediately upon request.

4 Results:

4.1 Were there really no patient dropouts? Please report whether any participants were excluded and provide reasons for exclusions based on the criteria. This information should be presented at the beginning of the results section.

Response: Based on our analysis, there were indeed no patients who withdrew midway. *It can be attributed to the following factors:* ***First,*** *the fixed weekly schedule for PICC maintenance provided participants with repeated and flexible opportunities to complete the survey during their routine clinic visits, thereby minimizing the risk of incomplete responses****. Second****, prior to the study, the research team dedicated substantial effort to standardizing survey procedures and communicating with patients in clear, accessible language. This ensured that participants could provide fully informed consent based on a thorough understanding of the study.* ***Finally,*** *the consistent and familiar environment of the vascular access clinic, along with the established trust between patients and the regular nursing staff, contributed to a supportive and cooperative atmosphere, facilitating continued participation. (Thank you for your suggestion. We have provided a brief analysis at the beginning of the results section.)*

We have excluded three patients with stroke combined with tumor disease due to their own movement disorders.

4.2 Include a flowchart showing the inclusion and exclusion of participants.
Response: Thank you sincerely for your suggestion. Based on the inclusion and exclusion criteria, we have presented the following flowchart. If it is not feasible, we kindly request further guidance

4.3 Improve the clarity of the tables and narrative. Emphasize key findings in the text for better understanding.

Response: We are grateful for the valuable suggestion. Based on this comment, we have revised the relevant paragraphs and incorporated the key findings from the univariate analysis to strengthen the manuscript. We would appreciate any further clarification should additional modifications be necessary.
5 Discusion

5.1 Numerous cancer-related studies have investigated kinesiophobia. Why did you choose to include literature on coronary and orthopedic conditions instead? Please revise these sections and use more relevant references.

Response: We thank the reviewer for this insightful comment. We agree that comparing our findings to studies on other cancer populations is more appropriate. In response, we have revised the discussion by removing comparisons with coronary and orthopedic conditions and have instead incorporated relevant references from oncology literature to provide a more focused context for our findings.
5.2 If you choose to divide the discussion into subsections, you must support each section with more comprehensive literature. However, while this format may provide clarity for the methods, it disrupts the continuity—particularly in the paragraph discussing regression results. Consider revising your discussion style.

Response: We sincerely appreciate the reviewer's insightful observation on the structure of our discussion. The discussion has been thoroughly revised in response, with the aim of enhancing its coherence and depth. We would be thankful for any further guidance the reviewer might offer, should any part require further refinement.
5.3 The studies you cite lack information on sample characteristics. Reporting findings without clarifying the patient populations involved is confusing and disrupts the coherence of the discussion. Please ensure contextual consistency.

Response: We thank the reviewer for this important observation. We have now revised the discussion to include relevant details (e.g., incidence of kinesiophobia) for the cited references to ensure a more coherent and meaningful comparison with our findings. And we further searched for relevant literature and read it thoroughly, adding comparisons and references to some of the literature.

For example, adding the following content to the discussion section：

*Notably, the observed prevalence is elevated compared to a report on breast cancer patients from Turkey (30.8%) [30], and it parallels a study on kinesiophobia conducted among postoperative breast cancer patients in Poland (40.8%-42.8%) [31]. This discrepancy with the Turkish study may be partly attributable to differences in sample characteristics. Our study encompassed a heterogeneous mix of solid tumor patients at various disease stages, whereas the cited study focused specifically on breast cancer survivors, a population that may have distinct rehabilitation experiences and support systems.*

*For the oncology patient, pain sources are multifactorial [36] (e.g., catheter stimulation, tumor infiltration, neuropathies), necessitating a sophisticated nursing response. It is worth noting that in our sample, which included patients with diverse cancer types (e.g., gastrointestinal, lung) and treatment regimens, the experience of pain would be inherently varied. Future research with larger samples could stratify by cancer diagnosis to determine if specific populations, such as those with bone metastases who are at higher risk of pathological fractures [37-38], exhibit disproportionately higher levels of kinesiophobia.*

**Reviewer 2**

On a general assessment, the topic under discussion proves to be noteworthy from multiple perspectives. The emerging data and core arguments possess the potential to fill a significant gap in the current literature. However, for the work to achieve its full impact, a deeper analysis of certain key aspects and a clearer establishment of its methodological framework are essential. In summary, this study has a strong foundation and, with careful revision, can leave a more robust and lasting impression in the academic field.

Response: We are truly grateful to the reviewer for their generous comments regarding the strong foundation of our study and its potential impact. We also sincerely appreciate the insightful guidance on how to enhance the work. The recommendation to perform a deeper analysis and to more clearly establish the methodological framework is well-received. Our team will diligently undertake the careful revision suggested to improve the robustness and clarity of the manuscript.

1 A literature review is present, but the gap is not clearly specified.

Response: We thank the reviewer for this critical observation. We agree that a clearly defined research gap is essential. In response, we have thoroughly revised the introduction section, try to explicitly state the research gap. We have now clearly articulated that while kinesiophobia has been studied in other patient groups, there is a lack of focused research on tumor patients with PICC, especially one that employs a theoretical framework to investigate the influencing factors. The modifications can be found on page 4-5 of the revised manuscript.

If the changes are not suitable, we kindly request further clarification from you. Thank you very much.

2 How HAPA theory was integrated into the study (e.g., variable selection) is not explained.

Response: Thank you for your pieces of advice. The selection of the HAPA model is justified in the introduction's fourth paragraph. We consider kinesiophobia to be a specific stage, dynamic and unhealthy behavior. This model is particularly suited to our research context as it effectively explains the progression from motivational intentions to volitional action in health behaviors. A key rationale for its adoption is its emphasis on the volitional, or actional, phase of change—a process critically mediated by factors such as self-efficacy, coping strategies, and action planning. Consequently, the model directly informed our choice of measurement constructs, thus ensuring our variable selection is theoretically grounded. Guided by this model, we have selected key constructs for measurement, including self-efficacy, risk perception, outcome expectations, and behavioral intention.

3 A clear research hypothesis should be added.

Response: Thank you for your pertinent suggestions. We have referred to previous publications in this journal and it seems that there is no specific requirement to write a hypothesis section. So, it is indeed our negligence in this regard. Can we make the assumption as follow: ①The study hypothesized that key constructs from the HAPA model, including lower exercise self-efficacy, higher risk perception, and lower exercise intention, would be significantly associated with higher levels of kinesiophobia; ②We hypothesized that a longer PICC indwelling time is associated with a lower incidence of kinesiophobia, as an enhanced understanding of the catheter may subsequently reduce exercise-related fears.

4 Missing data management is not explained.

Response: We thank the reviewer for raising this point. We are pleased to clarify that our data collection process yielded a complete dataset with no missing values for any of the analyzed variables. Should our interpretation be inaccurate, we would be grateful for further clarification.

5 Both ‘binary logistic regression’ and ‘multiple linear regression’ are mentioned – this is inconsistent.

Response: Thank you very much for your reminder. We made a mistake in writing the statistical methods section. We have made revisions in the manuscript.

***Statistical methods***

*SPSS 26.0 was used for data processing. Pearson correlation analysis was used between kinesiophobia and various variables, independent sample t-test or chi-square test was used for univariate analysis, multiple linear regression analysis was used for multivariate analysis.*

6 It should be stated whether regression assumptions were tested.

Response: We thank the reviewer for this crucial methodological suggestion. We agree that testing the regression assumptions is fundamental to ensuring the validity of our results. In response, we have added a detailed statement in the "Statistical Analysis" subsection of the Methods, describing how we verified the key assumptions of linear regression, including linearity, independence, normality, homoscedasticity, and the absence of multicollinearity. The revisions can be found on “Statistical methods” section.

*A multiple linear regression model was employed to identify the factors associated with kinesiophobia scores.* ***Prior to the final analysis, the underlying assumptions of linear regression were thoroughly examined. The linearity between continuous independent variables and the dependent variable was assessed visually using partial regression plots and was found to be satisfactory. The independence of residuals was confirmed by a Durbin-Watson statistic close to 2. The normality of the residuals was verified using a histogram and a normal Q-Q plot. Homoscedasticity (homogeneity of variance) was evaluated by plotting the standardized residuals against the standardized predicted values, which revealed no evident pattern. Finally, the absence of multicollinearity was ensured, as all variance inflation factor (VIF) values were below 10*** *(Only one VIF=5.1, the rest are below 5)****.***

7 The inclusion of variables in the model should be justified.

Response: We sincerely thank the reviewer for this insightful comment. We appreciate the opportunity to clarify this point. The selection of variables included in our analytical model was carefully considered and primarily guided by the Health Action Process Approach (HAPA) theoretical framework, complemented by clinical relevance and preliminary univariate analyses.

Core constructs from the HAPA model, such as Exercise Self-Efficacy, Risk Perception, and Exercise Intention/Outcome Expectancies, were included based on their established roles within the model's motivational and volitional phases of health behavior change.

Clinically relevant factors like Pain and Catheterization Duration were incorporated due to their significant associations identified in preliminary analyses and their direct relevance to the PICC care context.

The Medical Coping Modes Questionnaire and the Social Support Rating Scale were included as they represent critical contextual and moderating factors that may influence the fear-avoidance pathway, even if not central to the HAPA model itself.

8 The discussion section is quite long and repetitive.

Response: We sincerely thank you for your valuable suggestions. We have thoroughly revised the Discussion section accordingly. Given the extensive nature of the revisions, we kindly invite you to review the updated manuscript. We would be grateful for any further guidance you may have.

9 It contains a summary rather than an interpretation of the findings.

Response: We sincerely thank you for your valuable suggestions. We have thoroughly revised the Discussion section accordingly. Given the extensive nature of the revisions, we kindly invite you to review the updated manuscript. We would be grateful for any further guidance you may have.

10 Concrete recommendations based on the findings: pain and risk perception management in the early stages, self-efficacy-enhancing education programmes in the later stages.

Response: We sincerely thank the reviewer for this constructive suggestion. We have now integrated these concrete, staged recommendations into the revised manuscript. Specifically, the 'Conclusion' section explicitly proposes a phased nursing care plan: prioritizing pain and risk perception management immediately after PICC insertion, followed by structured self-efficacy-enhancing education programs in the later stages of catheter care. We hope these revisions meet the reviewer's expectations. We would be grateful for any further guidance should additional refinements be necessary to strengthen our recommendations.

11 A stronger nursing-focused discussion should be provided.

Response: In response to the valuable feedback, we have thoroughly revised the discussion to provide a stronger nursing-focused perspective. We have striven to articulate the clinical implications of our findings for nursing practice more explicitly. We would be grateful for any further guidance to ensure these modifications fully meet the journal's standards.

12 Terminology should be standardised.

Response: Thanks a lot. We also noticed this when making modifications and made some optimizations to the terminology, hoping to meet the requirements

13 Sentence structure should be simplified.

Response: Thanks a lot. We also noticed this when making modifications and made some optimizations to the terminology, hoping to meet the requirements

14 Patients' cancer types may also contribute to the development of kinesiophobia. It would be appropriate to add information about patients' disease duration and knowledge about cancer.

Response: We sincerely thank the reviewer for this insightful and valuable suggestion. We fully agree that patients' cancer types, disease duration, and knowledge about cancer are potentially significant factors influencing kinesiophobia, and incorporating them would undoubtedly enrich the analysis.

We sincerely apologize that, due to an oversight in the initial study design, data on specific cancer types and detailed disease duration were not systematically collected in the present study. We deeply regret this limitation and acknowledge that it constitutes a constraint on our current findings.

In response to this shortcoming and the reviewer's astute observation, we have explicitly stated this limitation in the revised manuscript's "Limitations and Future Directions" section. Furthermore, we plan to address this gap in our subsequent research. We intend to employ a longitudinal design and utilize Structural Equation Modeling (SEM) to explore the complex mechanisms of kinesiophobia more comprehensively, and we will ensure that cancer type, disease duration, and cancer knowledge are included as key variables in that future work.

We are truly grateful for the reviewer's guidance, which has been crucial in shaping our future research agenda.

15 References to studies on kinesiophobia related to different cancers could be added.

Response: After thorough reading, we have found that some of the references are very suitable for citation. Thank you very much for your guidance.

***We greatly appreciate the efficient, professional and rapid processing of our paper by your team. If there is anything else we should do, please do not hesitate to let us know.***

***Once again, thank you very much for your marvelous comments and suggestions.***

Sincerely,

Rong Li, Xiaohua Zhu, Yan Wu, Xiaozhu Qiao, Ying Yang, Fang Chen

Email：lirong19900916@163.com
