# Supplementary material for: Analysis of the current status and influencing factors of kinesiophobia in tumor patients with peripherally inserted central catheter: A cross-sectional study
Source: PLoS One. 2026 Feb 4;21(2):e0332064. doi: 10.1371/journal.pone.0332064 (PMC12872018; doi:10.1371/journal.pone.0332064)
Supplement: Attachment — Submitted filename: renamed_da9e8.docx [file pone.0332064.s004.docx]

Dear EDITOR,

Firstly, I would like to congratulate the authors on their work. I have suggested a few minor revisions in the form of notes in some parts of the article. I assessed its improved state with the previous revision. In addition, a few concrete suggestions regarding the impact of the study on nursing care could be added to the conclusion or discussion section. Thank you.

Asst Proff Arzu Nurdaş
