# Supplementary material for: Analysis of the current status and influencing factors of kinesiophobia in tumor patients with peripherally inserted central catheter: A cross-sectional study
Source: PLoS One. 2026 Feb 4;21(2):e0332064. doi: 10.1371/journal.pone.0332064 (PMC12872018; doi:10.1371/journal.pone.0332064)
Supplement: Attachment — Submitted filename: Response to Reviewers (Minor Revision).doc [file pone.0332064.s005.doc]

**Dear Editor and Reviewers,**

We sincerely thank the editor and reviewers for the opportunity to revise our manuscript and for their valuable, constructive feedback. We have carefully considered all comments and are pleased to resubmit a revised version. All modifications made in response to the feedback are highlighted in the manuscript with track changes. We hope our revisions are now satisfactory and that the manuscript is much improved for publication.

1.This sentence is getting off topic. ”Chemotherapy is required in approximately 72.6% of cancer cases”

**Response:** Thanks for pointing out that the sentence regarding the prevalence of chemotherapy was diverting from the core narrative. Upon reviewing, we indeed identified a problem of logical discontinuity. We had changed as follow: *For patients requiring chemotherapy, the peripherally inserted central catheter (PICC) has become the most widely used central venous access in China due to its minimally invasive nature, avoidance of repeated punctures, and convenience for safe medication*. Should the modifications fall short, we would appreciate your further guidance.

2.What re these conditions and how do they differ from physical activity disorder?

**Response:** We thank the reviewer for this critical observation, which has helped us clarify a key methodological point. We agree that the original terms "contraindications to exercise" and "physical activity disorders" were ambiguous and potentially overlapping. We have revised the exclusion criteria in the Methods section to precisely differentiate between them:

“Absolute contraindications to physical activity” now refers to specific, medically prohibitive conditions (e.g., unstable cardiovascular disease).

“Severe neurological or musculoskeletal disorders” is used to describe conditions that cause substantial physical limitation. The rationale for this exclusion is to ensure that a high score on the kinesiophobia scale truly reflects a fear of movement rather than an inability to move due to organic pathology, thereby preserving the construct validity of our primary outcome.

Guided by your comment, we believe these clarifications strengthen the methodological rigor of our study. Please see the updated as follows:

*①Have absolute contraindications to physical activity* (e.g., unstable cardiovascular disease, acute systemic infection, or other conditions where exercise is medically prohibited as determined by the treating physician).

*②Have severe comorbid conditions that could independently limit survival or confound the assessment* (e.g., severe heart failure [NYHA Class III/IV], end-stage renal disease on dialysis, or severe immunocompromised state).

*③Have a documented history of mental illness or cognitive impairment* (e.g., dementia, schizophrenia) that would impede the ability to provide informed consent or accurately report symptoms.

*④Have severe neurological or musculoskeletal disorders that substantially limit voluntary limb movement* (e.g., paralysis, severe Parkinson's disease, or advanced osteoarthritis preventing basic arm or leg movement), making the assessment of kinesiophobia related to fear rather than physical incapacity unreliable.

Looking forward to your further guidance.

3.p<0,001 if bonferoni correction was made it should be stated or found to be highly significant.

**Response:** We thank the reviewer for raising this important methodological point regarding multiple comparisons. In our univariate analysis (Table 1), the P-values reported for multi-category variables (e.g., education level, placement duration) were derived from the initial omnibus tests (e.g., one-way ANOVA), not from subsequent pairwise comparisons. As our primary aim at this stage was variable screening for the regression model (using a threshold of P < 0.05), and we did not conduct or report post-hoc pairwise comparisons between individual groups, a Bonferroni correction was not required. To prevent any ambiguity, we have revised the ‘Statistical methods’ section to clarify this approach explicitly. We now state: “*Variables yielding a P-value < 0.05 in these initial omnibus tests were considered candidates for multivariate analysis. We did not perform post-hoc pairwise comparisons; therefore, a Bonferroni correction was not applied*.” We hope this clarification satisfactorily addresses the reviewer's concern.

Should any part require further refinement, we warmly welcome your expert advice and would deeply appreciate your additional feedback.

4. A few concrete suggestions regarding the impact of the study on nursing care could be added to the conclusion or discussion section.

**Response:** We sincerely thank the reviewer for this constructive suggestion to enhance the practical impact of our study. To maintain conciseness in the Discussion, we have consolidated the concrete, actionable suggestions into the Conclusion section. In direct response, we have revised the Conclusion section to incorporate concrete, actionable recommendations for nursing practice. These are structured around a phased intervention model derived from our findings: We now added:

…ultimately improving the long-term prognosis and quality of life of tumor patients. “*To translate these findings into practice, we propose three concrete steps for nursing care:1.Implement Routine Screening: Integrate the TSK-11 or a brief screener into PICC maintenance visits to systematically identify patients with kinesiophobia. 2. Adopt a Phased Intervention Model: Early phase (≤1 month): Prioritize pain management and risk perception correction through standardized visual education and multidisciplinary analgesia. Later phase (>1 month): Focus on building exercise self-efficacy via graded goal-setting (e.g., progressive walking plans), peer support, and activity diaries. 3.Equip Frontline Nurses: Develop concise, evidence-based training tools (e.g., brief videos, checklists) to enable nurses to deliver these interventions efficiently in routine practice.”*

We believe these specific additions bridge the gap between research findings and clinical application, directly addressing the reviewer's recommendation.

***We greatly appreciate the efficient, professional and rapid processing of our paper by your team. If there is anything else we should do, please do not hesitate to let us know.***

***Once again, thank you very much for your marvelous comments and suggestions.***

Sincerely,

Rong Li, Xiaohua Zhu, Yan Wu, Xiaozhu Qiao, Ying Yang, Fang Chen

Email：lirong19900916@163.com

2025.12.16
